# Decay of *Enterococcus faecalis, Vibrio cholerae* and MS2 Coliphage in a Laboratory Mesocosm Under Brackish Beach Conditions

**DOI:** 10.3389/fpubh.2019.00269

**Published:** 2019-09-24

**Authors:** Ananda Tiwari, Ari Kauppinen, Tarja Pitkänen

**Affiliations:** The Finnish Institute for Health and Welfare, Kuopio, Finland

**Keywords:** bathing water quality, survival rate, enterococci, *Vibrio* spp., F-specific coliphage, the Baltic Sea, beach environment

## Abstract

Enterococci are fecal indicator bacteria (FIB) used for monitoring the microbial quality of bathing water. However, the reliability of health protection by monitoring FIB is questioned. This study evaluated the decay pattern of *Enterococcus faecalis* in beach environment and compared it with decay patterns of the pathogen *Vibrio cholerae* and the virus indicator MS2 coliphage. Tests were done in an experimental mesocosm simulating natural Nordic summer daylight conditions with and without the aquatic plant *Myriophyllum sibiricum*. The decay of the spiked test microbes (*E. faecalis, V. cholera*, and MS2) was enumerated up to 27 days from two coastal bathing water mesocosms with subtidal sediment. *E. faecalis* and *V. cholerae* exhibited non-linear biphasic decay patterns and were detected upmost toward the end of the experiment in the water, sediment, and vegetation. The gene copies of *V. cholerae* dropped to a minimum by days 6–8 but then the numbers increased back up to nearly the spiked level. The MS2 coliphage was detected only up to 8–10 days into the experiment solely in the water where a log-linear decay pattern was seen. The test microbe, sample origin (water, sediment or vegetation) and, as determined for *E. faecalis*, the enumeration method (culture-based or qPCR) affected the decay pattern. *E. faecalis* decayed faster in water than in sediment and vegetation. Positive correlations between culturable *E. faecalis* counts with *V. cholerae* gene copies and MS2 counts were recorded on the first few days of the experiment. This study demonstrated the important role of water, sediment and vegetation regarding the partitioning of pathogens and fecal indicators in coastal environment. The enumeration of the enterococci counts alone was not sufficient for predicting the numbers of pathogens such as *Vibrio* spp. in bathing water. The growth of *Vibrio* spp. in the Baltic Sea deserves more attention and might require water quality monitoring to be applied for these pathogens in the coming years due to the predicted rise in sea surface temperature favoring *Vibrio* spp. growth. Further, different decay patterns observed between MS2 and enterococci emphasize the need for and importance of a viral indicator in assessing water quality more comprehensively.

## Introduction

Enterococci are official fecal indicator bacteria (FIB) in many countries for determining the bathing water quality and monitoring with the intention to protect swimmers from exposure to enteric pathogens (1, 2). An ideal FIB has a similar decay rate to enteric pathogens (3). However, the wide taxonomic range of microbes in the water relevant to the health of swimmers might have a different response toward environmental stress and growth factors such as pH values, solar radiation, salinity, predation, temperature and nutrients (4). Further, studies provide evidence that enterococci and enteric pathogens may persist longer in aquatic sediment and vegetation than in water (4, 5). In certain aquatic conditions, enterococci may grow in beach environments (5, 6) and enterococci originating from natural sources may pose unnecessary false positive alarms of a health risk. In contrast, the higher persistence of pathogens over FIB may jeopardize the health of beach users due to false negative alarms (4). Therefore, understanding the ecological interactions of different fecal indicators and pathogens with various biotic and abiotic factors found at bathing sites is important.

In a warming climate, in which surface water temperatures are rising, the characterization and predictions of health risks to swimmers are more important than ever (7). In the brackish Baltic Sea region, pathogenic members of the *Vibrio* bacterial genus are a concern because their proliferation benefits from a rise in the sea surface temperature (8–10). In recent history, the summertime heat waves of 2014 and 2018 have shown that a rise in surface water temperatures together with water nutrients and favorable wind conditions can lead to extensive occurrences of *Vibrio* spp. in the coastal waters of the Baltic Sea (11, 12). Particularly in the northern parts of the Baltic Sea, a subsequent rise of *Vibrio* spp. infections, mainly wound and ear infections, have been noted (11, 13) and pose a significant health risks to certain groups of people, namely the elderly, immunocompromised patients and persons with open wounds (14). Several *Vibrio* species with pronounced pathogenic potential (including the non-O1/non-O139 serotype of *Vibrio cholerae* and *Vibrio vulnificus, Vibrio parahaemolyticus*, and *Vibrio alginolyticus*) are known to occur in the Baltic Sea region (9–11, 13), and notifiable *V. cholerae* infections in Finland have been associated with severe manifestations of illnesses such as septicemia (11).

A rise in the bathing water temperature may have also indirect effects on microbial loads on beaches. High temperatures attract crowds to beaches for relief, especially if exceptional, extreme weather events occur (7). High temperatures may thus challenge beach management paradigms, and norovirus outbreaks have been associated with a sudden increase in the number of beach users (15). Furthermore, previous studies have detected viruses in surface water even when the FIB numbers were below the safe limit according to current monitoring protocols (15, 16). Such findings imply that FIB is insufficient for indicating the presence of enteric viruses in environmental waters; which justifies the need for introducing a virus indicator for fecal contamination to assess health risks of bathers (3, 17). Coliphages resemble human enteric viruses in their physical structure, composition, morphology, and survival characteristics in the environment and have been suggested as a potential virus indicator associated with human fecal contamination for monitoring bathing water (3). F-specific coliphage MS2 is one of the most commonly used virus indicators for water quality testing (3).

Almost every year by the end of the bathing season, bathing sites on the coast of the eastern Gulf of Bothnia (Baltic Sea) have experienced problems with overly high enterococci counts, without a known contamination source. Instead of fecal contamination, excess growth of aquatic vegetation has been noted at these bathing sites, while our earlier study identified enterococci species of mostly *Enterococcus faecalis* (18). Additionally, a knowledge gap has been noted concerning the survival and growth strategies of *Vibrio* spp. and especially *V. cholerae* as an emerging etiological agent for vibriosis in the temperate coastal bathing sites of the Baltic Sea as a result of climate change (19). In addition, more knowledge is needed on the use of MS2 coliphage as a virus indicator for fecal contamination in coastal environments.

This study evaluated the decay pattern of *E. faecalis*, one of the most dominant enterococci species in the human gut (20), in beach water, sediment and vegetation. The enterococci decay was compared with decay patterns of the pathogen *V. cholerae* and virus indicator MS2 coliphage in an experimental mesocosm simulating natural Nordic summer daylight conditions. Further, our objective was to investigate the role of aquatic vegetation in survival of *E. faecalis, V. cholerae* and MS2 coliphage in the beach environments.

## Materials and Methods

### Mesocosm Preparation

Water, sediment, and vegetation were collected from an official bathing site located on the western coast of Finland (Gulf of Bothnia, the Baltic Sea) and transported immediately after collection to the laboratory in cool boxes. Approximately 80 liters (l) of coastal water was collected in plastic containers. Similarly, coastal sediment weighing about 30 kg was collected from the subtidal zone and mixed well. Further, aquatic vegetation (*M. sibiricum*) of about 3 kg in weight was manually picked with roots from the bathing site. As illustrated in Figure S1; two 50 l aquariums (Kuopion Akvaariovalaisin, Kuopio, Finland) were filled to form the study mesocosms with an approximately 4 cm layer of sediment and then by adding water (~35 l) with a final volume ratio of sediment and water of 1:4 [following Badgley et al. (5)]. About 2 kg (wet weight) of *M. sibiricum* was planted in one aquarium. The mesocosm without the vegetation was coded BS and the mesocosm with vegetation as BSM (BS, Baltic Sea; M, *M. sibiricum*).

Mesocosms were exposed to identical artificial solar radiation simulating natural Finnish summer daylight conditions (19 h of daylight) using Sylvania Reptistar T8 full spectrum lamps (438 mm, ø 26 mm, 15 W, 6,500 K, UV-A 30%, UV-B 5%; Giant Valaisin, Kuopio, Finland). Both mesocosms were placed on a laboratory bench at ambient room temperature and covered to reduce evapotranspiration.

The mesocosms were fitted with identical aquarium pumps (Ismatec ISM-1079B, VAC-115/230 Ecoline, Wertheim, Germany) without a filter to create a continuous mix of water and to avoid an anoxic environment. All the metallic equipment which came into contact with the samples, i.e., mechanical stirrers, scissors, blades and spoons were autoclaved prior to use. Aquariums and pumps were cleaned with a soap-water and 5 mg/l chlorine solution and then rinsed with tap water (3 times) and then with distilled water (3 times), prior to use. Both mesocosms were fitted with a minimum and maximum thermometer for measuring the temperature (°C).

### Spike Preparation

After settling the sediment (for about 30 min), suspensions of *E. faecalis, V. cholerae* and MS2 were spiked into the BS and BSM mesocosms. The *E. faecalis* 13V1235-1 strain was isolated from a coastal bathing site during a high enterococci count event [(18); sampling site E]. The *V. cholerae* non-O1, non-O139 strain was isolated from a vibriosis patient in the 2014 heat wave on the western coast of Finland (11). Both strains were stored at −75°C or lower before use in this study. The strains of *E. faecalis* and *V. cholerae* were cultivated on tryptone soya agar medium (TSA) and a blood agar medium and incubated at 36°C for 48 and 24 h, respectively. Colony material was suspended in a phosphate buffer to reach an absorbance of 0.8 at 420 nm with the aim to spike about 3 × 10^8^ CFU of both *E. faecalis* and *V. cholerae* into the water of both mesocosms. The colony counts of *E. faecalis* and *V. cholerae* in the spikes were enumerated by spread plating on TSA and blood agar media, respectively.

An MS2 coliphage (NCTC 12487) was produced using an *E. coli* host (ATCC 15597) following the principles presented in ISO 10705-1 (1995). Chloroform (1:10 v/v) was used to extract phages from the solution. The aqueous phase was centrifuged at 5,000 g for 20 min at 4° C and then filtered through a 0.45 μm filter (Acrodisc, Pall Corporation). The phage stock solution was stored at 4° C until use. The estimated initial number of the MS2 coliphage was the same as the *E. faecalis* and *V. cholerae*, being approximately 3 × 10^8^ PFU for each mesocosm.

### Mesocosm Monitoring

The physico-chemical and microbiological water quality was monitored for both mesocosms over time from duplicate samples. Natural background numbers of the microbial targets were enumerated as duplicates from the mesocosm water, sediment and vegetation before adding the spike. The sampling was continued after adding the spike (sampling for the initial numbers was conducted 30 min after the spike) and then at first twice a day (0.5, 1, 1.5, 2, 2.5, and 3 days), then every second day (4, 6, 8 and 10 days) and finally before ending the experiment on days 13, 20 and 27. A multi-parameter analyzer (Multi 3430; WTW GmbH, Weilheim, Germany) was used for measuring the pH, dissolved oxygen (mg/l) and conductivity (μS/cm) of the water samples. Further, the turbidity (NTU) was measured from subsamples in the laboratory at a wavelength of 860 nm with a Turb 555IR spectrophotometer (WTW GmbH&Co. KG, Weilheim, Germany) and the chloride concentration (mg/l) was monitored using the mercuric thiocyanate method as described in the manufacturers manual (Method 8113; HACH Lange GmbH, Duesseldorf, Germany).

The workflow for each sampling event was as follows for both BS and BSM to avoid the unnecessary mixing of the mesocosm: (a) the temperature was recorded (b) water samples (~50 ml) were collected with a peristaltic pump to measure physical characteristics (c) water samples (10–1,000 ml in duplicate) were collected with a peristaltic pump for microbial analysis, (d) vegetation samples (only from BSM; about 1 g in duplicate) were collected with the help of a sterile rod and scissors, and (e) sediment samples (about 3 g in duplicate) were collected with the help of a sterile 2 ml micro-centrifuge tube. For processing the vegetation and sediment samples, a phosphate buffer at a ratio of 1:10 was used and the mixture was vortexed for 2 min (1,100 rpm), settled for 30 s and then the eluent was transferred into a clean tube for microbial analysis [the protocol modified from Whitman et al. (21)].

### Target Enumeration

The culturable counts of intestinal enterococci including *E. faecalis* were enumerated using membrane filtration according to the international standard method ISO 7899-2:2000 as described by Tiwari et al. (18). In brief, after filtration of multiple sample volumes, the membranes (GN6, Pall Life Sciences, Michigan, USA) were incubated on a Slanetz & Bartley medium (S&B, Oxoid Ltd. Basingstoke, Hampshire, England) at 36 ± 2°C for 44 ± 4 h. A range of sample volumes were used to produce 10–100 presumptive colonies per membrane. After counting the presumptive enterococci, the membrane was transferred on a preheated bile aesculin azide medium (BEA, Scharlau, Barcelona, Spain) and incubated at 44 ± 0.5°C for 2 h. A black or brown color formation on bacterial colonies on the BEA medium confirmed the colony belonged to the group of intestinal enterococci. The enterococci counts in the water were presented as CFU/100 ml and in sediment and vegetation as CFU/100 mg (wet weight).

The MS2 coliphage was measured using a culture-based method with a double agar layer (DAL) technique from 0.5 mL samples (22) using *E. coli* (ATCC 15597) as a host in two replicates. The MS2 counts in water were presented as PFU/100 ml and for the sediment and vegetation as PFU/100 mg (wet weight).

For molecular analyses, a subsample of water (50–300 ml) was concentrated onto 0.45 micrometer-pore-size, 47-mm-diameter polycarbonate membranes (Nuclepore, Whatman) and 0.35 ml of each vegetation and sediment eluent was directly transferred into a micro-centrifuge tube. The membranes and tubes were stored at −75°C or lower prior to nucleic acid extraction. The total DNA and RNA were extracted from the samples using the AllPrep DNA/RNA Mini Kit (Qiagen GmbH) as previously described (23). The RNA was further purified using the Ambion TURBO DNA-free DNase kit (Life Technologies, Grand Island, NY), and then complementary DNA (cDNA) was synthesized using the random hexamer primed Superscript III system for RT-PCR (Life Technologies). The total RNA was stored at −75°C or lower, while the cDNA and the DNA extracts were stored at −20°C until use.

The gene copy numbers of *Enterococcus* spp. and *Vibrio* spp. in the samples (including extraction and filtration blanks) were measured from cDNA and DNA extracts, and the *Vibrio cholerae* and Gram-negative bacteria gene copy numbers were measured from the DNA extract only. The qPCR assays were performed as previously described (23), by processing 8 μl of RNA in a cDNA synthesis (reverse transcription, RT). The primers and probes used in this study are listed in the Table S1. The targeted genes were 23S rRNA gene for *Enterococcus* spp. [Entero1 assay; (24)] 16S rRNA gene for *Vibrio* spp. (25), and the *ompW* gene was used for the *V. cholerae* (26, 27). Total bacterial numbers in the BS and BSM waters were evaluated by using an assay targeted to 16S rRNA gene of all Gram -negative bacteria (28). The qPCR reactions were performed using a QuantStudio 6 real-time PCR system (Applied Biosystems) in 20 μl volume using the TaqMan Environmental PCR Master Mix (Life Technologies) for *Enterococcus* spp., *V. cholerae* and Gram- assays and the Power Sybr PCR Master Mix (Life Technologies) for a *Vibrio* spp. assay, all with primers and probes at final concentrations 0.2 μM (IDT Technologies, Inc). The cycling conditions included 95°C for 10 min of enzyme activation and pre-denaturation followed by 40 cycles at 95°C for 15 s of denaturation and at 60°C for 60 s of annealing, except for *Vibrio* spp., for which a 64°C annealing temperature was used and a melt curve analysis of the PCR amplicons was performed. Standard curves were generated using artificial gene fragments (gBlocks, IDT Technologies, Inc.) containing the sequences for each of the targeted genes.

In qPCR, undiluted and 10 fold diluted cDNA and DNA samples were used as qPCR templates to detect PCR polymerase inhibition. For samples in which PCR inhibition was detected, qPCR data was generated using the results from diluted samples. Background signals, if detected in the filtration blanks, were subtracted from all the results to generate the final qPCR and RT-qPCR data per assay. The limit of detection (LOD) was set as 3 copies per reaction. The copy number per 100 ml of water and 100 mg of sediment and vegetation was calculated for those samples with values above the limit of quantification (LOQ) (i.e., as determined by the lowest value within the quantification range). The final qPCR, equivalent LOD (eLOD) and equivalent LOQ (eLOQ) values were calculated after taking into account the volume/mass of the processed sample, factors associated with the different processing steps of the RNA and DNA manipulations, and the dilutions used for each sample analyzed.

### Identification of Enterococci Colonies

Representative enterococci colonies grown on an S&B medium were identified with partial 16S rRNA gene sequencing as done in our previous study (18). Enterococci colonies isolated before the spike were identified from all the substrates (water, sediment and vegetation). Further, the enterococci colonies isolated from water samples after 1, 2, and 10 days from the spike, sediment samples after 6 and 20 days and vegetation samples after 4 and 10 days were identified. From the selected samples, the nucleic acid extracts of all or at least 10 enterococci colonies grown were amplified with universal bacteria primers 8F (5′-AGAGTTTGATCCTGGCTCAG-3′) and 787R (5′-CGACTACCAGGGTATCT AAT-3′) as described earlier by Ryu et al. (29). The quality of the sequences was checked and the consensus sequences from paired reads were made with the BioEdit, bioinformatics software (30). Finally, the enterococci species were identified with the NCBI Blast database (31).

### Decay Rate

The decay characteristics of the targets were tested with GInaFit (Geeraerd and Van Impe Inactivation Model Fitting Tool) freeware add-in for Microsoft Excel 2010 (32). Models were selected based on the lowest root mean sum of the standard errors (RMSE), and the highest R^2^ value as done earlier by Kauppinen and Miettinen (33). The details of the microbial decay model equations are shown in Supplementary Material (Microbial decay and decay rate).

GInaFit does not support modeling an erratic type of decay data. For that condition, the reduction equation was calculated with the following log-linear decay model equation [as done by Badgley et al. (5) and Anderson et al. (4)]:

$$\begin{array}{l} Log_{10}\frac{N_{t}}{N_{0}}=-kt \end{array}$$

where k = the decay rate, N_t_ = the target count at time t days, N_0_ = the target count 0-day, t = the time (in days) when the target count is N_t_.

### Data Analyses

All the data was log_10_ transformed and expressed as the log_10_ CFU/PFU/GC per 100 ml for the water and per 100 mg for the sediment and vegetation. Where applicable, mean of duplicate samples BS and BSM was used. The data analysis was done with SPSS (IBM SPSS Statistics for Windows, Version 25.0, IBM Corp., Armonk, NY). In this study, the significance was compared at a 95% confidence level, if not mentioned otherwise. The median numbers of the targets and water quality parameters between the two mesocosms were compared using Wilcoxon signed-rank test. The relation of culturable *E. faecalis* with other targets was calculated using Spearman's rank correlation test.

The significance of the differences of the *k*-values was tested as done by Green et al. (34), by calculating the lower and upper confidence intervals with the following equation:

$${\text{Confidence interval (CI) }=\text{k ± Standard Error (SE)}}^{*}\text{α}$$

where the SE was obtained from GInaFiT tool and the α value was obtained from a Student's *t*-table on (n−2) degrees of freedom at a 95% confidence interval. The *k*-values were significantly different if the confidence intervals of two *k*-values did not overlap.

## Results

### Water Quality in Two Mesocosms

The effect of vegetation on the properties of water was calculated by comparing the physico-chemical properties and total numbers of Gram-negative bacteria in the water without vegetation and with vegetation (BS and BSM, respectively). Table 1 shows the mean and median of the measured parameters of water in both mesocosms. The vegetation significantly reduced the median turbidity of the water in the BSM compared to the BS samples (*p*-value 0.008; Table 1). The mean oxygen and chloride concentrations were slightly higher in the water of vegetated BSM mesocosm water than in the BS water, but the difference was not statistically significant (Table 1). The water temperature, pH and electric conductivity were not affected by vegetation. The water temperature increased in both mesocosms during the course of experiment from about 19°C to 23°C. The numbers of Gram-negative bacteria measured as gene copies per 100 ml and indicating the total number of bacteria in water, were significantly higher in the BS compared to BSM (*p*-value 0.007; Table 1).

**Table 1:** Physico-chemical parameters and the number of Gram-negative bacteria in the water of mesocosms without vegetation (BS) and with vegetation (BSM).

**Parameters**	**Mean (SD)**		**Median**		**Significance (Wilcoxon test; *p*-value)**
	**BS**	**BSM**	**BS**	**BSM**	
Turbidity (NTU)	4.8 (3.6)	2.9 (2.7)	4.1	1.7	0.008
O_2_ (mg/l)	9.2 (0.6)	9.4 (0.6)	9.2	9.4	0.062
Chloride (mg/l)	2,200 (210)	2,000 (100)	2,220	2,000	0.073
Temperature (during sampling) (°C)	22.3 (1.7)	22.4 (1.4)	22.7	22.9	0.477
pH	7.7 (0.2)	7.6 (0.1)	7.7	7.6	0.450
Electric conductivity (μS/cm)	8,400 (240)	8,300 (140)	8,300	8,300	0.677
Gram-negative bacteria (log_10_ gene copies/100 ml)	7.7 (0.3)	7.2 (0.4)	7.8	7.5	0.007

*SD, standard deviation*.

### Target Detection and Decay

**Figure 1:**
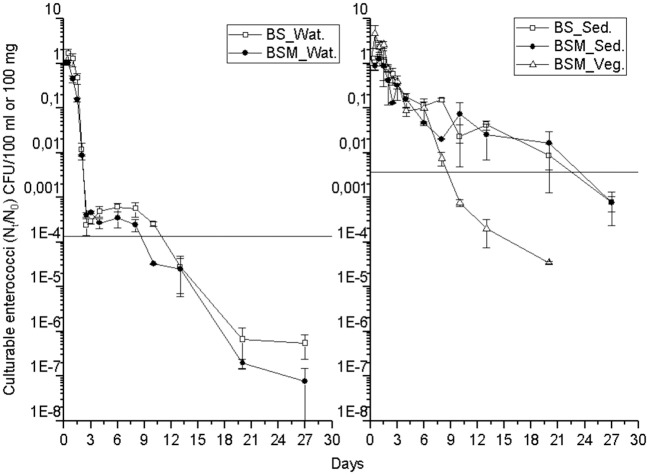
The mean number (N_t_/N_0_) of culturable enterococci over time within the water (Wat.), sediment (Sed.) and vegetation (Veg.). The vertical bar of a sampling point indicates the range of duplicate samples. The horizontal line shows the background number of the target before adding the spike. For backround counts of the targets (see Tables 2, 3). BS—unvegetated mesocosm and BSM—vegetated mesocosm.

**Figure 2:**
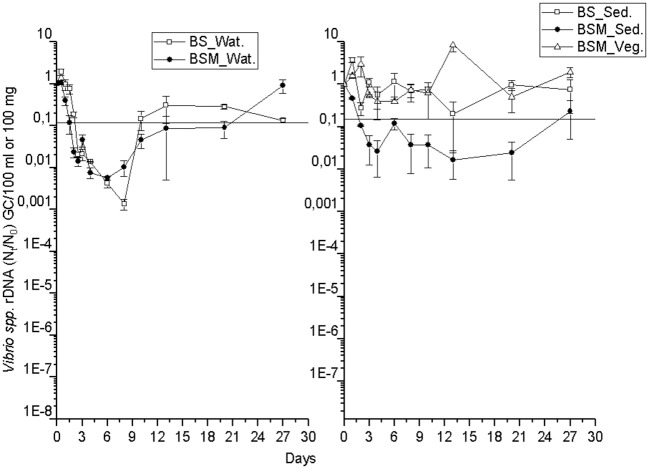
The mean number (N_t_/N_0_) of *Vibrio* spp. rDNA gene copies over time within the water (Wat.), sediment (Sed.) and vegetation (Veg.). The vertical bar of a sampling point indicates the range of duplicate samples. The horizontal line shows the background number of the target before adding the spike. For backround counts of the targets (see Tables 2, 3). BS—unvegetated mesocosm and BSM—vegetated mesocosm.

**Figure 3:**
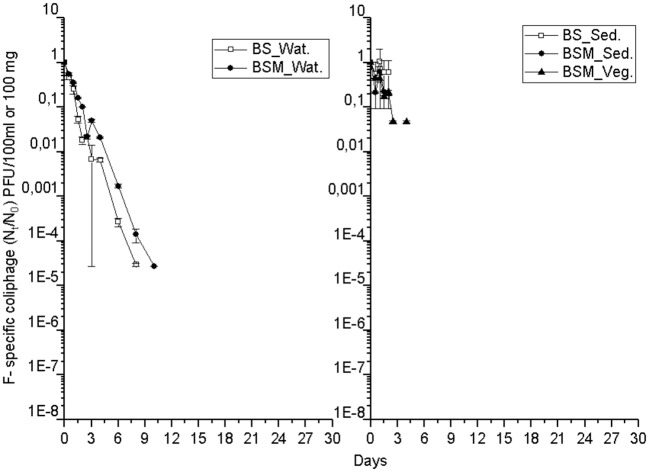
The mean number (N_t_/N_0_) of MS2 coliphages over time within the water (Wat.), sediment (Sed.) and vegetation (Veg.). For backround counts of the targets (see Tables 2, 3). BS—unvegetated mesocosm and BSM—vegetated mesocosm.

The detection and decay rate of the targets was determined for each target microbe, as well as for the substrate from which the target was sampled, and also for both enumeration methods of enterococci (culture-based and molecular). Culturable enterococci were detected up to the end of the experiment in the water and sediment, but only up to the 20th day in the vegetation (Figure 1). Further, when using molecular assays, the rRNA marker was more frequently detected than the rDNA marker (Figures S2 and S3), indicating the higher 23S rRNA transcript copy numbers in the ribosomes of the viable *Enterococcus* spp. cells as compared to the copy numbers 23S rRNA gene in the *Enterococcus* spp. genome (23). However, culturable enterococci were detected more frequently than rRNA or rDNA *Enterococcus* markers, the result related to differences between the studied sample volumes and different limits of detection between the methods. *Vibrio* spp. genus-specific markers (rDNA, rRNA) were detected up to the end of the experiment in the water, sediment and vegetation (Figure 2 and Figure S4). The *V. cholerae* species-specific marker was less frequently detected than the genus-specific markers (Figure S5). Among the different substrates, *Vibrio* spp. was more frequently detected in the water than in the sediment or vegetation. MS2 coliphage was detected only by the 8th−10th days of the experiment in the water, and only during the 3 first days of the experiment in the sediment and vegetation (Figure 3).

**Figure 4:**
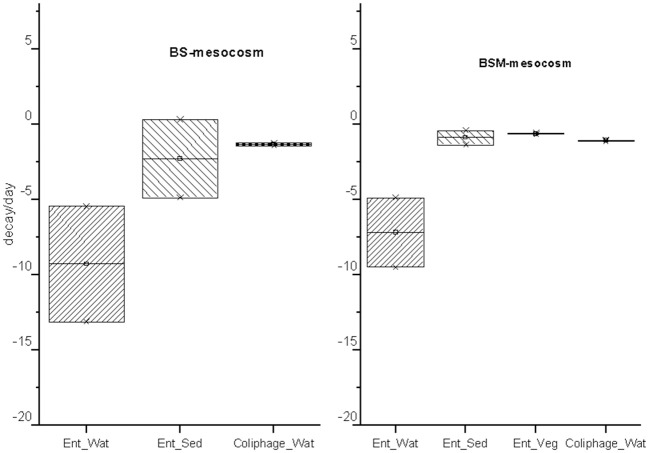
The decay rate (k)/day and standard error (SE) obtained from GInaFit model with upper and lower confidence intervals for intestinal enterococci (Ent.) and MS2 coliphages (coliphage) in water (Wat.), sediment (Sed.) and vegetation (Veg.). The box indicates the decay rate confidence interval of the decay rate (CI) = k ± SE*α_(N−2)degreeoffreedom_ at a 95% confidence level.

The decay characteristics of each target were calculated using the GInaFiT tool and with a classical log-linear model and are shown in Tables 2 and 3, respectively. The culturable enterococci followed the biphasic or biphasic decay model with a shoulder with the first rapid decay phase before the delayed decay phase, in all the substrates (water, sediment and vegetation) (Figure 1; Table 2). The decay rate (*k*-value) of the culturable enterococci in the first rapid decay phase was 7.0 and 9.7/day in water of vegetated (BSM) and unvegetated mesocosm (BS), respectively. The culturable enterococci numbers reached the background level after the 8th−13th days of the experiment. The decay rate of culturable enterococci in the sediments of the BS and BSM was 2.6 and 0.8 per day in the first phase and 0.3 and 0.1 per day in second phase of the biphasic decay, respectively (Figure 4; Table 2). In the vegetation of BSM, the enterococci decay was log linear with the rate of 0.8 per day. The culturable enterococci had a significantly higher decay rate in water than in sediment and vegetation, as the upper and lower confidence interval of the k-value did not overlap (visual inspection; Figure 4).

**Table 2:** Log-linear and nonlinear GInaFiT decay model results for culturable enterococci and MS2 coliphages.

**Mesocosm**	**Substrate**	**Log-Linear model and parameters**						**Best fitted non-linear model and parameters**					
		**Log_**10**_(N_**nat**_) / 100 ml or 100 mg**	**t_**bac**_ (days)**	**Log_**10**_(N_**0**_) / 100 ml or 100 mg**	**Kmax ± SE (days^**−1**^)**	**R^**2**^**	**RMSE**	**Best fitted model**	**k_**max1**_ ± SE (day^**−1**^)**	**k_**max2**_ ± SE (day^**−1**^)**	**SL ± SE**	**R^**2**^**	**RMSE**
**Culturable enterococci**													
BS	W	1.9	13	5.7	−0.8 ± 0.2	0.60	1.2	B. S.	−9.7 ± 4.0	−0.2 ± 0.1	1.42 ± 0.2	0.97	0.4
BSM	W	1.9	10	5.9	−0.8 ± 0.2	0.70	1.1	B. S.	−7.0 ± 1.7	−0.3 ± 0.1	1.13 ± 0.2	0.99	0.2
BS	S	2.1	27	4.8	−0.3 ± 0.0	0.94	0.3	B. S.	−2.6 ± 2.7	−0.3 ± 0.0	1.93 ± 0.7	0.96	0.3
BSM	S	1.7	27	4.7	−0.3 ± 0.0	0.88	0.33	B.	−0.8 ± 0.3	−0.2 ± 0.0	na	0.90	0.3
BSM	V	3.2	10	5.5	−0.8 ± 0.1	0.96	0.29	–	–	–	–	–	–
**MS2 coliphage**													
BS	W	0.2	8	4.5	−1.3 ± 0.1	0.97	0.57	–	–	–	–	–	–
BSM	W	ND	na	4.6	−1.1 ± 0.1	0.98	0.2	–	–	–	–	–	–
BS	S	ND	na	0.8	na	na	na	–	–	–	-	–	–
BSM	S	ND	na	0.7	na	na	na	–	–	–	–	–	–
BSM	V	ND	na	1.3	−0.5 ± 0.1	0.94	0.3	–	–	–	–	–	–

*BS, mesocosm without vegetation; BSM, mesocosm with vegetation; W, water; S, sediment; V, vegetation; N_nat_, background count before spike; N_0_, count at the zero hour (just after spike); t_bac_, time required for target counts to reach the background level; B.S., biphasic with shoulder; B, biphasic; D, 1–log reduction time; 4D, 4-log reduction time; R^2^, goodness of fit; RMSE, root mean sum of the standard errors; SE, standard error; SL, shoulder length; ND, not detected; na, not applicable*.

**Table 3:** Decay rates (log-linear) of *Enterococcus* spp., *Vibrio* spp. and *V. cholerae* genetic markers.

**Mesocosm**	**Substrate**	**log10(N_**nat**_) GC/100 ml or 100 mg**	**t_**bac**_**	**log_**10**_(N_**0**_) GC/100 ml or 100 mg**	**log_**10**_(N_**min**_) GC/100 ml or 100 mg**	**t_**min**_**	**k_**min**_ (day^**−1**^)**
***Enterococcus*** **spp. (rDNA)**							
BS	Water	2.88	10–13	5.96	3.48	10	−0.19
BSM	Water	3.08	8–10	6.01	3.52	8	−0.19
BS	Sediment	ND	>0	2.96	na	na	na
BSM	Sediment	ND	>0.5	1.81	na	na	na
BSM	Vegetation	ND	>4	3.34	na	4	−0.03
***Enterococcus*** **spp. (rRNA)**							
BS	Water	4.57	13	7.55	3.17	20	−0.24
BSM	Water	4.45	20	7.08	2.91	20	−0.22
BS	Sediment	6.06	27	7.73	4.93	27	−0.08
BSM	Sediment	6.57	6	7.09	5.00	27	−0.1
BSM	Vegetation	6.73	13	8.31	5.61	27	−0.1
***Vibrio*** **spp. (rDNA)**							
BS	Water	5.42	2.5	6.71	3.84	8	−0.19
BSM	Water	5.58	2	7.21	4.95	6	−0.38
BS	Sediment	3.08	2	3.71	2.87	13	−0.15
BSM	Sediment	3.49	3	4.73	2.98	13	−0.08
BSM	Vegetation	3.82	>27	4.55	4.16	4	−0.13
***Vibrio*** **spp. (rRNA)**							
BS	Water	4.4	>27	9.25	5.97	8	−0.41
BSM	Water	6.74	>27	9.32	7.08	6	−0.36
BS	Sediment	8.7	4	8.79	7.09	20	−0.13
BSM	Sediment	8.94	3	8.81	6.87	8	−0.22
BSM	Vegetation	9.46	3	9.52	8.98	20	−0.04
***V. cholerae*** **(DNA)**							
BS	Water	ND	>27	5.65	2.37	8	−0.41
BSM	Water	ND	>27	5.88	2.85	6	−0.51
BS	Sediment	ND	>1	2.16	na	na	na
BSM	Sediment	ND	>1	ND	na	na	na
BSM	Vegetation	ND	>27	2.44	2.07	3	−0.13

*BS, mesocosm without vegetation; BSM, mesocosm with vegetation; N_nat_, background count before spike; t_bac_, time required for target counts to reach the background level; k_min_, decay rate calculated with the equation Log_10_ (N_t_/N_0_) = –kt; where N_t_ was the lowest target count over the course of experiment after adding the spike and t was the respective day when the target count was the lowest; ND, not detected; na, not applicable. The k-value for the rDNA of Enterococcus spp. and V. cholerae in sediment and vegetation was not available due to their low detection frequency*.

The MS2 coliphage followed the log-linear decay model in water of both the mesocosms. The counts remained continuously higher in the BSM water than in BS water (Figure 3). Further, a higher decay rate of the MS2 coliphage was noted in the BS water than in the BSM water, k-values were −1.3 and −1.1 per day, respectively, in the BS and BSM water (Figure 4; Table 2).

GInaFiT was not able to identify the decay model for the *Enterococcus* spp. genetic markers (rRNA, rDNA). However, from the visual inspection (Figures S2 and S3), the biphasic decay of rRNA in water and sediment and an almost log linear pattern in vegetation was seen. The decay of the *Enterococcus* spp. genetic markers (rDNA, rRNA) was slower than the decay of culturable enterococci counts in all substrates. The markers exhibited a higher decay rate (k-value) in water (0.19–0.24/ day) than in sediment (0.08–0.1/ day) and vegetation (0.03–0.1/ day) (Table 3). It took 13 and 20 days after the spike in BS and BSM water, respectively, for *Enterococcus* spp. rRNA to reach the background level (Figure S2).

Additionally, for genetic markers of *Vibrio* spp. (rDNA, rRNA) and *V. cholerae*, GInaFiT was not able to identify the decay model. On visual inspection, a biphasic decay pattern was seen as the gene copies dropped rapidly up to day 6–10 but later increased back nearly up to the spiked level (Figure 2, Figures S4 and S5). The log-linear decay rate of all *Vibrio* markers was lower in vegetation than in water or sediment with the exception of the rDNA marker in the sediment of the BSM water (Table 3).

### The Relationship of *Vibrio cholerae* and MS2 With Culturable Enterococci

The relation between the enumerated numbers of culturable enterococci with other microbial targets in the water is shown in Table 4. Culturable enterococci had a strong positive correlation with *Enterococcus* spp. rDNA and rRNA markers. Overall, *Vibrio* spp. and *V. cholerae* markers had weak correlations with culturable enterococci. However, the correlation was strongly positive (ρ = 0.81–0.92) in the first 4 days after the spike. The culturable enterococci in the sediment and vegetation had a strong correlation with culturable enterococci in the water (Table 4). The correlation of the MS2 coliphage with culturable enterococci was strong in the first 6–8 days of the experiment (ρ > 0.85). There was also strong relation between culturable enterococci counts and *Enterococcus* spp. rDNA and rRNA gene copy numbers in water (correlation coefficients ρ = 0.83–0.87, Table 4).

**Table 4:** Comparing Spearman's rho correlation coefficient (ρ) between culturable enterococci with other microbial targets.

	**Targets**	**Substrates**	**27 days**	**First 4 days**
Culturable enterococci (Water)	*Enterococcus* spp. rDNA	Water	0.83^**^	0.87^**^
	*Enterococcus* spp. rRNA	Water	0.85^**^	0.86^**^
	MS2 coliphage	Water	0.87^^**^^#^^	0.90^**^
	*Vibrio* spp. rDNA	Water	0.28^*^	0.81^**^
	*Vibrio* spp. rRNA	Water	0.23	0.88^**^
	*V. cholerae* DNA	Water	0.33^*^	0.92^**^
	Culturable enterococci	Sediment	0.86^**^	0.73^**^
	Culturable enterococci	Vegetation	0.94^**^	0.80^**^

# The correlation was measured only until the MS2 coliphage was detected (up to 8–10 days).

** The correlation is significant at the 0.01 level (2-tailed).

* *The correlation is significant at the 0.05 level (2-tailed)*.

## Discussion

This study identified biphasic with a shoulder, biphasic and log-linear decay patterns of culturable enterococci, *Vibrio* spp. and MS2 coliphage, respectively, in coastal bathing water. Culturable enterococci decayed rapidly in water for up to 2.5 days, before a slower decay phase. The identified slightly higher decay rate of culturable enterococci in this study than noted in earlier studies with coastal waters (4, 35, 36) could be due to differences in the experimental settings. While, our study used an environmental enterococci strain under 21 h of continuous artificial solar radiation each day at room temperature (21–23°C), Craig et al. (35) focused on the effect of the temperature, and did not mention the solar radiation, while Anderson et al. (4) used enterococci from dog feces, waste water and soil inoculum in their experiment on rooftop of a building with an open-air greenhouse, and Zhang et al. (36) spiked targets directly from treated sewage.

The detected biphasic decay of *Enterococcus* spp. molecular markers (rDNA, rRNA) in water confirmed the recent findings by Ahmed et al. (37). Although the molecular marker decay seems to be slower than the decay of culturable enterococci counts, the strong correlation between the culturable counts and gene copy numbers generated using qPCR and RT-qPCR were found. This result indicates potential usability of molecular methods as alternatives to culture based methods for water quality monitoring.

The biphasic decay of microbes could be due to the target strain heterogeneity; various strains might have different decay rates (38). In our study, this is not a probable explanation as a single *E. faecalis* strain was used as a spike, and all the identified isolates from the samples before the spike belonged to the same species. Thus, the cause of the initial rapid decay of enterococci could be due to a rapid inactivation after a sudden change of environmental conditions such as the solar radiation, nutrient availability, predation, salinity or water temperature, but could be also related to initial growth phase and high density of spiked bacterial cells (39). Similar decay is expected to take place after a fecal contamination incidence into bathing waters and must be taken into consideration when interpreting bacterial indicator results. Later on, the introduced bacteria might start to cope better with the prevailing environment, so after an adaption phase their decay rate might be lower as was noted in the present study.

Additionally, *Vibrio* spp. and *Vibrio cholerae* genetic markers exhibited biphasic decay patterns. The good persistency of *Vibrio* spp. in water was as expected, since these bacteria are autochthonous to coastal waters (8, 10, 40, 41). The results presented herein correspond to one earlier study in which Zhang et al. (36) demonstrated the good persistence of *Vibrio* OTUs using high-throughput sequencing in a laboratory experiment. In the present study, the gene copies of *V. cholerae* dropped to a minimum by days 6–8 but then the numbers increased back nearly up to the spiked level. It is obvious that the current bathing water quality monitoring against fecal contamination (1) is not protecting bathers from *Vibrio* infection risks. In the present study, the observed lack of correlation between the genetic markers of *Vibrio* spp. and *V. cholerae* with culturable enterococci after the first 4 days highlights the deficiency of current monitoring practice. However, further studies are needed to investigate if *Vibrio* spp. counts could be enumerated from bathing waters using culture-based methods (42). It is possible that such counts could relate to counts of culturable enterococci better than the *Vibrio* marker gene copy numbers. The Nordic aquatic environment is rich in carbon and phosphorous and is favorable to the proliferation of *Vibrio* spp. (9). The pronounced proliferation of pathogenic *Vibrio* spp. in coastal waters is expected to cause greater public health concerns in future years due to global warming and the subsequent rise in sea surface temperatures (8–10, 43). *Vibrio* spp. have short generation times (<10–30 min) at their optimum growth temperature (23–25°C) and can respond rapidly to changing environmental conditions (10, 44). Therefore, for health risk management purposes, early-warning modeling tools such as Vibrio Viewer [https://e3geoportal.ecdc.europa.eu/SitePages/Vibrio%20Map%20Viewer.aspx; (13)] are useful. Further, the determination of *Vibrio* species from the bathing waters might become as a relevant effort to carry out, especially in cases of suspected waterborne infections.

The log-linear decay pattern of the MS2 coliphage resembled the decay patterns reported in earlier laboratory studies on F-specific coliphages, adenoviruses, and polyomaviruses in coastal waters (37, 45). Further, Craig et al. (35) reported a relatively slower decay rate of somatic coliphages in coastal water mesocosms and found that the temperature affected the decay rate. MS2 is one of the most UV resistant phage and used commonly as a conservative virus surrogate (46). In the present study, vegetation might have sheltered MS2 as the decay rate was slower in the BSM water than in the BS water. In fact, MS2 counts correlated with the culturable enterococci in the first few days of the experiment, which makes sense in the mesocosm mimicking natural environment. The decay of culturable MS2 calls for further studies to investigate its survival mechanisms. More information is needed for comparison if similar behavior is seen with viral pathogens.

In our study, bacterial targets persisted longer in sediment and vegetation than in water (except for *Vibrio* spp. rRNA). This better persistency detected herein has been reported also in several earlier studies in which the availability of nutrients, protection from UV radiation and predation, and lowered temperature have been identified as factors enhancing the survival (4, 5, 17, 35, 36, 40, 47). Further, the higher persistency of surface attached microbes in sediment or vegetation than in water may be due to the surface biofilm protecting microbes from external stress factors (48). Schets et al. (49) detected norovirus only in the sediment but not in the water at a bathing site during a bathing water outbreak. In our study herein, the poor detection frequency of the MS2 coliphage in sediment (12%) and vegetation (13%) could be due to the methodological limitations (47). Here, we used the same protocol for the separation of the MS2 coliphage and enterococci from sediment and vegetation, which was originally developed for enterococci (21).

Vegetation can play further complex roles in aquatic systems and lead to changes in water turbidity, pH, temperature, and predator species (50), of which the turbidity change was noted in the present mesocosm study. Furthermore, in our study, the oxygen concentration was slightly higher in BSM than in BS water, potentially due to photosynthesis of vegetation. In general, due to the settling effect, vegetation in water may reduce suspended solid, leading to less turbid waters. However, due to the shedding effects, temperature and UV exposure may decrease enabling better microbial survival when vegetation is present. However, after growth phase, the decomposing vegetation might have an opposite effect on water quality. Such changes can have multiple effects on the decay rate of the studied targets. In our experiment, vegetation cover could have effect on enterococci decay as we observed a slightly higher decay rate of enterococci in BS water than in BSM water in the first phase of the biphasic decay pattern. Also the MS2 coliphage was detected in a shorter time (8 days) in BS water than in BSM water (10 days) and the vegetation seemed thus to reduce the decay rate in water. Consistent with our findings, also Badgley et al. (5) demonstrated that vegetation reduced the decay rate of enterococci in fresh water in a similar experimental setting. In contrast, however, Kleinhrinz et al. (48) demonstrated that vegetation increased the decay rate of *Escherichia coli, Salmonella*, and *Shigella*, and they argued that the targets rapidly attached to vegetation and thus recorded a lower count from the water column.

In addition to the inactivation, the rapid reduction of microbial targets in the water could be due to sedimentation and attachment to the surface sediments (35, 51). The attachment of the targets to turbid suspended particles and their settling and deposition at the bottom of the mesocosm could thus have an effect on the initial rapid decay rate. In our study, culturable enterococci counts in the sediment and vegetation related strongly to culturable enterococci counts in the water. We did not notice the significant difference between the median value of enterococci counts in BS and BSM water. However, the total number of Gram-negative bacteria was higher in BS than in BSM water. These findings could partly be explained due to a partitioning effect. While the total numbers of bacterial targets were not different in the mesocosms, the BSM environment had three substrates (water, sediment, and vegetation), but the BS environment only had two (water and sediment). Thus, benthic sediment and aquatic vegetation could work as a sink and source of microbes like FIB and probable human pathogens (6). The FIB sink (due to sedimentation) in the benthic sediment and vegetation could contribute to the lower count of FIB in the water during regulatory monitoring of the microbial quality of bathing water, and their probable resuspension can complicate the interpretation of the results of water quality monitoring. The current microbial water quality monitoring protocol does not account for the microbial quality of the sediment and vegetation at bathing sites. The contamination and decay are simultaneous and continuous processes at each bathing site.

## Conclusion

This study demonstrated the partitioning of pathogens and fecal indicators in coastal waters between water, sediment and vegetation. It also indicated that the decay of microbes was different in water than in sediment in presence or absence of vegetation. The current bathing water monitoring protocol does not account for the microbial content of sediment and vegetation at bathing sites and thus possibly underestimates the actual enterococci counts during the regulatory monitoring of water. Furthermore, enumerating only the enterococci does not seem enough for the prediction of free-living pathogens such as *Vibrio* spp. Finally, the different decay patterns observed between MS2 and enterococci emphasize the need for and importance of a viral indicator for assessing water quality more comprehensively.

## Data Availability Statement

All datasets generated for this study are included in the manuscript/Supplementary Files.

## Author Contributions

All the authors participated in the project design, the execution of the experimental work, data analysis and manuscript writing. AT calculated the data and drafted the initial version of the manuscript. AK was incharge of the MS2 work. TP supervised the work.

### Conflict of Interest

The authors declare that the research was conducted in the absence of any commercial or financial relationships that could be construed as a potential conflict of interest.
