# Supplementary material for: Decay of Enterococcus faecalis, Vibrio cholerae and MS2 Coliphage in a Laboratory Mesocosm Under Brackish Beach Conditions
Source: Front Public Health. 2019 Sep 24;7:269. doi: 10.3389/fpubh.2019.00269 (PMC6771298; doi:10.3389/fpubh.2019.00269)
Supplement: Supplementary file 1 [file Data_Sheet_1.pdf]

Supplementary Material

Decay of *Enterococcus faecalis, Vibrio cholerae* and MS2 coliphage in a laboratory mesocosm under brackish beach conditions

Tiwari Ananda[1*], Kauppinen Ari[1], Pitkänen Tarja[1]

[1] The National Institute for Health and Welfare, P.O Box 95, 70701 Kuopio, Finland

*** Correspondence:** Email: ananda.tiwari@thl.fi, Telephone: +358442535468

1 Supplementary Table

Table S1 | The oligonucleotide sequences used in the (RT)-qPCR assays in the study.

Assay name	Target species	Sequence 5'to 3'	Chemistry	Length (bp)	Reference
Entero1	*Enterococcus* spp.	ECST748F: AGAAATTCCAAACGAACTTG<br>ENC854R: CAGTGCTCTACCTCCATCATT<br>GPL813TQ: TGGTTCTCTCCGAAATAGCTTTAGGGCTA	TaqMan	92	Ludwig & Schleifer (2000)
Vibrio	*Vibrio* spp.	567F: GGCGTAAAGCGCATGCAGGT<br>680R: GAAATTCTACCCCCCTCTACAG	Sybr Green	114	Thompson et al. (2004)
Vcho	*V. cholerae*	ompW-F: TCAATGATAGCTGGTTCCTCAAC<br>ompW-R: CGATGATAAATACCCAAGGATTGA<br>ompW-P: TGGTATGCCAATATTGAAACAACG	TaqMan	126	Garrido-Maestu et al., (2014), Garrido-Maestu et al., (2016)
Gram-	Gram-negative bacteria	Gram-F: GGGTTAAGTCCCGCAACGA<br>Gram-R: CATTGTAGCACGTGTGTAGCCC<br>Gramneg-P: TGACGTCAANTCATCATGNCCCTTANG	TaqMan	155	(Kärkkäinen et al., 2010)

References

1. Garrido-Maestu A., Chapela M.-J., Penaranda E., Vieites J.M. and Cabado A.G. (2014). In-house validation of novel multiplex real-time PCR gene combination for the simultaneous detection of the main human pathogenic vibrios (*Vibrio cholerae, Vibrio parahaemolyticus*, and *Vibrio vulnificus*). *Food control.* 37(0) pp.371-379. doi: 10.1016/j.foodcont.2013.09.026
2. Garrido-Maestu A., Lozabo- Leon A., Rodriguez- Souto R.R., Vieites- Maneiro R, Chapela M-J, Cabado A.G. (2016). Presence of pathogenic *Vibrio species* in fresh mussels harvested in the southern Rias of Galicia (NW Spain). *Food Control* (59) 759- 765. doi: 10.1016/j.foodcont.2015.06.054
3. Kärkkäinen, P., Valkonen, M., Hyvärinen, A., Nevalainen, A., Rintala, H. (2010) Determination of bacterial load in house dust using qPCR, chemical markers and culture. *J. Environ. Monit.* 12(3), 759-768. doi: 10.1039/B917937B.
4. Ludwig W. and Schleifer K-H. (2000). How quantitative is quantitative PCR with respect to cell counts? *System. appl. Microbiol.* (23) 556-562. doi: 10.1016/S0723-2020(00)80030-2
5. Thompson J.R., Randa M.A., Marcelino L.A., Tomita-Mitchell A., Lim E., Polz M.F. (2004). Diversity and dynamics of a North Atlantic coastal *Vibrio* community. *Appl. Environ. Microbiol.* 70 (7) 4103-4110.doi: 10.1128/AEM.70.7.4103-4110.2004

2 Supplementary Material

Microbial decay and decay rate

In the controlled laboratory experiment, the decay model of a single strain population is generally a log-linear reduction model (Bevilaxqua et al. 2015) and is expressed in $\log_{10}$ reduction (Equation I).

$$Log_{10}\ Reduction\ (LR) = Log_{10}N_0 - Log_{10}N_t \qquad \text{Equation (I)}$$

In case of complex environmental setting and with multiple target strains (population) the decay model may not be linear (Bevilacqua et al. 2015). Biphasic decay model is one common example of nonlinear decay model; which represents two phases of decay, having two different decay slopes (k_{max1} and k_{max2}) in decay equation. The following equation (Equation II) describes the biphasic decay model:

$$Log_{10}(N) = Log_{10}(N_0) + \log\,[f.\,e^{k\,max_1 t} + (1-f)e^{k\,max_2 t}] \qquad \text{Equation (II)}$$

Similarly, biphasic model with the shoulder is described by the following equation (Equation III; Bevilaxqua et al. 2015):

$$Log_{10}(N) = Log_{10}(N_0) + \log_{10}\ [f.\ e^{-k_{max1}.\,t}\ \frac{e^{k_{max1}.SL}}{1+(e^{k_{max1}.SL}-1)e^{-k_{max1}\,.t}} + (1-f).\ e^{-k_{max2}.t}.$$

$$\{\frac{e^{k_{max1}.SL}}{1+(e^{k_{max1}.SL}-1).e^{-k_{max1}\,.t}}\}^{\frac{k_{max2}}{k_{kmax1}}}\ ] \qquad \text{Equation (III)}$$

Here f is the group of population which has inactivation rate k_{max1} and (1- f) is the group of population which has inactivation rate k_{max2}.

The Weibull model of bacterial decay assumes the lethal events are probabilities and that the corresponding survival curves are cumulative forms of a distribution of the lethal event. The following equation (Equation IV) describes the Weibull decay model (Van Boekel, 2002):

$$Log_{10}(N) = Log_{10}(N_0) - ((\frac{t}{delta})^p) \qquad \text{Equation (IV)}$$

Where delta is the time required to attain a 1- log reduction in cell count. P is dimensionless and linked with geometrical shape of the curve.

References

1. Bevilacqua A., Speranza B., Sinigaglia M. and Corbo M.R. (2015). A focus on the death kinetics in predictive microbiology: benefits and limits of the most important models and

some tools dealing with their application in foods. *Foods*, 4, 565-580. doi: 10.3390/foods4040565
2. Van Boekel M. A. J. S. (2002). On the use of the Weibull model to describe thermal inactivation of microbial vegetative cells. *Int. J. Food Microbiol.*, 74 (1-2), 139-159. doi: 10.1016/S0168-1605(01)00742-5

3 Supplementary Figures

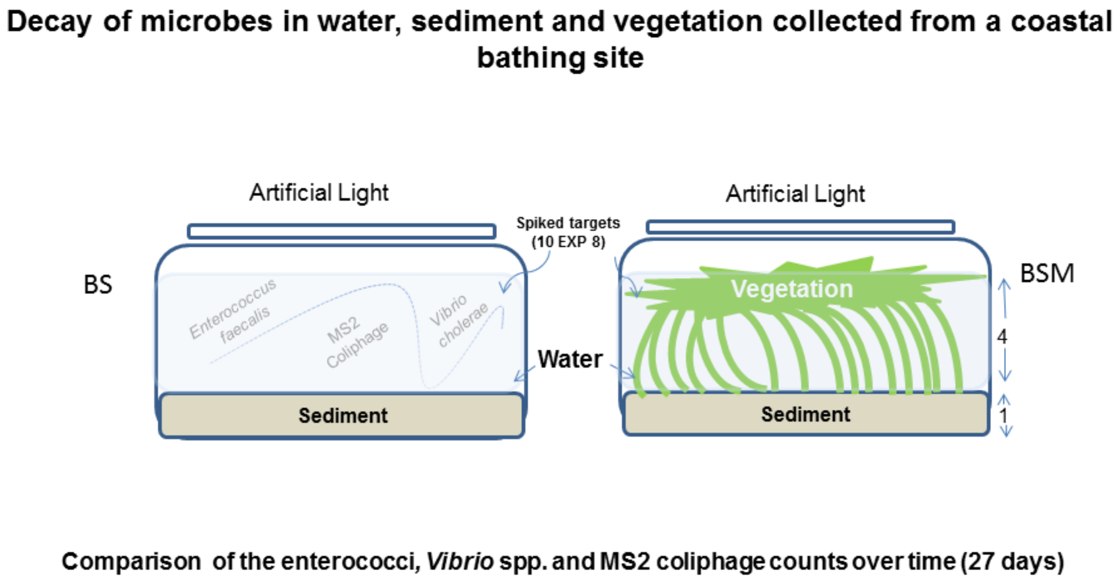

Figure S1. A graphical layout of experimental setup.

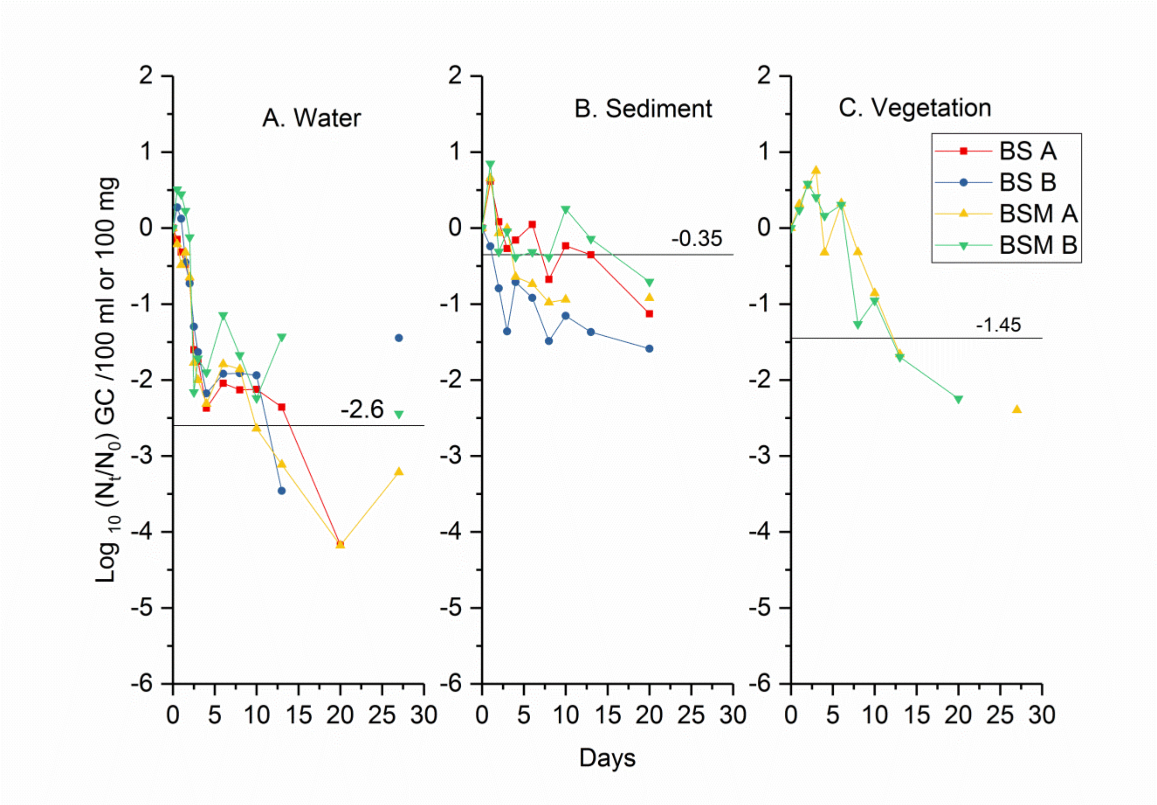

Figure S2. The number of *Enterococcus* spp. rRNA copies (N_t/N_0) in water, sediment and vegetation presented as duplicates (A and B) in mesocosms BS (without vegetation) and BSM (with vegetation). The horizontal line shows the background numbers of rRNA copies before adding the spike.

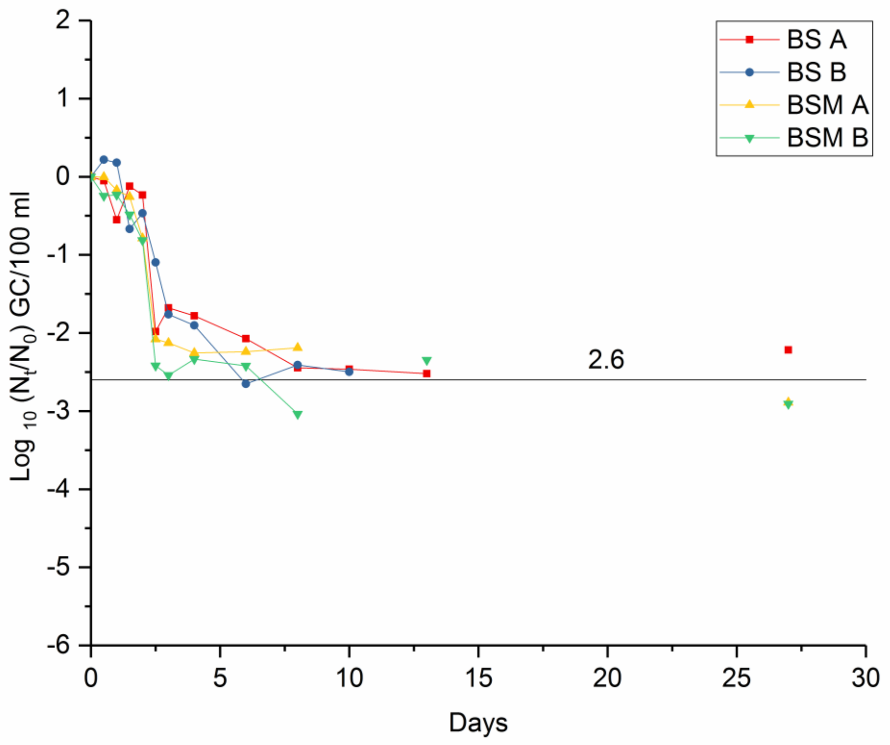

Figure S3. The number of *Enterococcus* spp. rDNA gene copies (N_t/N_0) in water presented as duplicates (A and B) in mesocosms BS (without vegetation) and BSM (with vegetation). The horizontal line shows the background number of rDNA gene copies before adding the spike. The samples collected from sediment and vegetation did not contain rDNA gene copies above the background (*i.e.*, the results were below the detection limit).

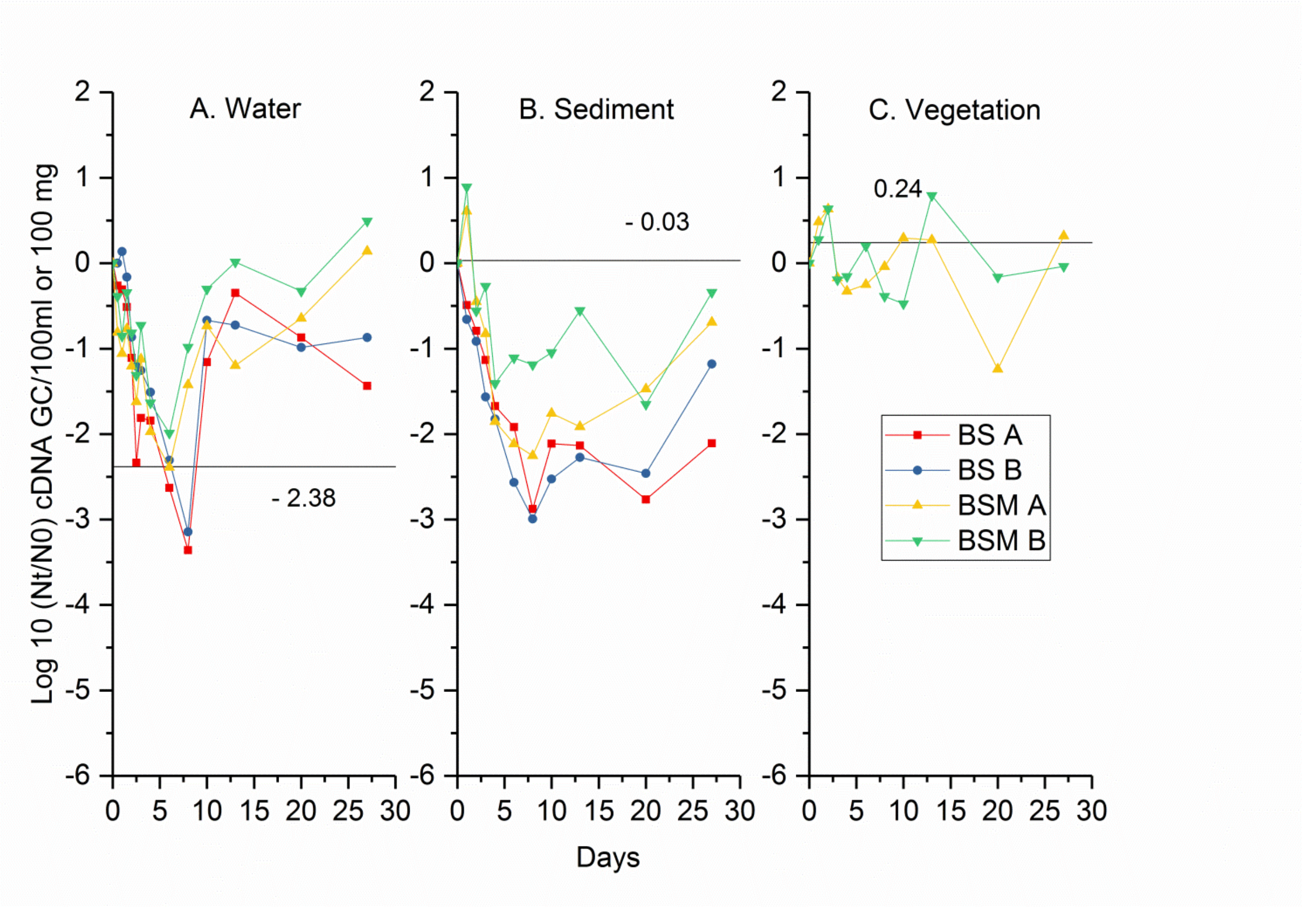

Figure S4. The number of *Vibrio* spp. rRNA copies (N_t/N_0) in water, sediment and vegetation presented as duplicates (A and B) in mesocosms BS (without vegetation) and BSM (with vegetation). The horizontal line shows the background numbers of rRNA copies before adding the spike.

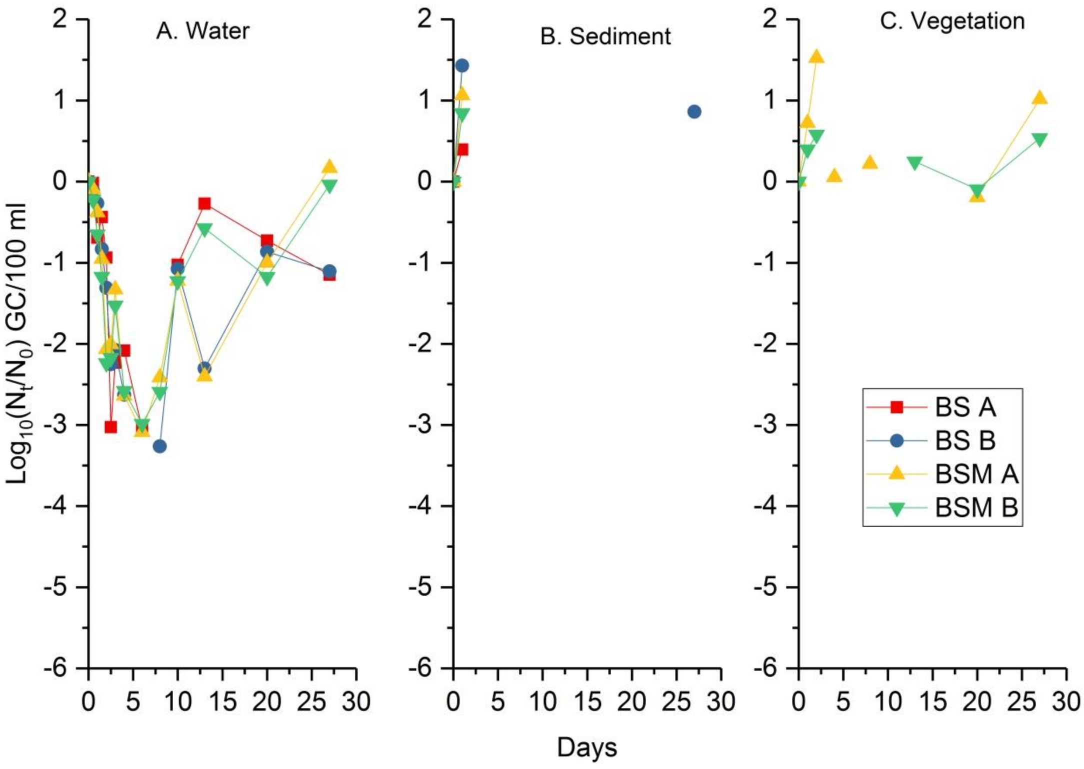

Figure S5. The number of *V. cholerae ompW* gene copies (N_t/N_0) in water, sediment and vegetation presented as duplicates (A and B) in mesocosms BS (without vegetation) and BSM (with vegetation). *V. cholerae ompW* gene was not detected in the background samples (before spike).
